# *Pseudomonas aeruginosa* OprF plays a role in resistance to macrophage clearance during acute infection

**DOI:** 10.1038/s41598-020-79678-0

**Published:** 2021-01-11

**Authors:** Malika Moussouni, Laurence Berry, Tamara Sipka, Mai Nguyen-Chi, Anne-Béatrice Blanc-Potard

**Affiliations:** Laboratory of Pathogen-Host Interactions (LPHI), CNRS-UMR5235, Université de Montpellier, Montpellier, France

**Keywords:** Immunology, Microbiology

## Abstract

While considered an extracellular pathogen, *Pseudomonas aeruginosa* has been reported to be engulfed by macrophages in cellular and animal models. However, the role of macrophages in *P. aeruginosa* clearance in vivo remains poorly studied. The major outer membrane porin OprF has been recently shown to be involved in *P. aeruginosa* fate within cultured macrophages and analysis of an *oprF* mutant may thus provide insights to better understand the relevance of this intramacrophage stage during infection. In the present study, we investigated for the first time the virulence of a *P. aeruginosa oprF* mutant in a vertebrate model that harbors functional macrophages, the zebrafish (*Danio rerio*) embryo, which offers powerful tools to address macrophage–pathogen interactions. We established that *P. aeruginosa oprF* mutant is attenuated in zebrafish embryos in a macrophage-dependent manner. Visualization and quantification of *P. aeruginosa* bacteria phagocytosed by macrophages after injection into closed cavities suggested that the attenuated phenotype of *oprF* mutant is not linked to higher macrophage recruitment nor better phagocytosis than wild-type strain. Using cultured macrophages, we showed an intramacrophage survival defect of *P. aeruginosa oprF* mutant, which is correlated with elevated association of bacteria with acidic compartments. Notably, treatment of embryos with bafilomycin, an inhibitor of acidification, increased the sensibility of embryos towards both wild-type and *oprF* mutant, and partially suppressed the attenuation of *oprF* mutant. Taken together, this work supports zebrafish embryo as state-of-the-art model to address in vivo the relevance of *P. aeruginosa* intramacrophage stage. Our results highlight the contribution of macrophages in the clearance of *P. aeruginosa* during acute infection and suggest that OprF protects *P. aeruginosa* against macrophage clearance by avoiding bacterial elimination in acidified phagosomes.

## Introduction

The environmental bacterium *P. aeruginosa* is an opportunistic human pathogen responsible for a variety of acute infections and is a major cause of mortality in chronically infected cystic fibrosis patients. Numerous reports have emphasized that the extracellular pathogen *P. aeruginosa* can enter host cells, resulting in a phase of intracellular residence, which can be of importance in addition to the classical extracellular infection. An intracellular stage of *P. aeruginosa* within cultured epithelial cells has been known for a long time^1–3^ and advanced imaging methods have allowed to track bacteria within epithelial cells^4^. More recently, *P. aeruginosa* has been also localized within cultured macrophages^5–7^. The intramacrophage fate of the bacteria has revealed vacuolar escape of *P. aeruginosa* and macrophage death driven by intracellular bacteria, most likely linked to cytosolic location of bacteria^8^. Bacterial factors involved in this intramacrophage step have been recently investigated^6,8^. MgtC and OprF have been uncovered as bacterial factors involved in the intramacrophage survival of *P. aeruginosa*^6,7,9^. Our work recently established that MgtC and OprF modulate the transcription of type III secretion system (T3SS) genes. T3SS, and more specifically its ExoS effector, play a main role in the intramacrophage life of *P. aeruginosa*, allowing internalized bacteria to escape phagosomes and promote macrophages lysis^8^. Consistent with the effect of OprF on T3SS genes transcription, OprF modulated the production of the T3SS PcrV cap protein and the secretion of ExoT and ExoS toxins^10,11^.

OprF is a major outer membrane porin involved in maintenance of cell structure, outer membrane permeability, environmental sensing, adhesion, biofilm formation and virulence^12,13^. Besides regulating the secretion of T3SS effectors, OprF also modulates the production of the quorum-sensing-dependent virulence factors pyocyanin, elastase, lectin PA-1L, and exotoxin A^10^. Accordingly, in the *oprF* mutant, production of the quorum-sensing signal molecules is reduced or delayed^10,12^. The virulence of *oprF* mutant is reduced in an invertebrate model organism, the nematode *C. elegans*^10^. Moreover, a reduction in the cytotoxicity was observed for the *oprF* mutant on various cultivated mammalian cell lines, including epithelial cells and macrophages^7,10^.

The role of macrophages in vivo in internalization and early clearance of *P. aeruginosa* remains poorly studied, as shown with the controversial results regarding the contribution of mouse lung alveolar macrophages in *P. aeruginosa* infection^14,15^. In the present study, we explored the behavior of an *oprF* mutant in connexion with intramacrophage survival during *P. aeruginosa* acute infection in a non-mammalian vertebrate animal model. Zebrafish (*Danio rerio*) embryo is a model of choice for studying interactions between pathogens and host innate immune system, due to its optical transparency that allows the analysis of infections in real time using fluorescent microorganisms^16,17^. Moreover, zebrafish innate immune system is closely related to the one of mammals and transgenic reporter lines are available to visualize innate immune cells^16,17^. This model has therefore been highlighted as a suitable model to address the interaction between bacteria and macrophages^18–20^. *P. aeruginosa* has been shown to be phagocytosed by macrophages upon acute infection in zebrafish embryos^21,22^, thus allowing to study in vivo the bacterial factors involved in *P. aeruginosa* survival into macrophages and evaluate their importance in the outcome of infection. In the present study, we first investigated the behavior of *oprF* mutant strain in vivo upon infection of zebrafish embryo, before moving to further analysis with cultured macrophages. Our results highlight the contribution of macrophages in the clearance of *P. aeruginosa* during acute infection and support a role of OprF in the ability of *P. aeruginosa* to avoid localization in acidified phagosomes and resist elimination by macrophages.

## Results

### In the *Danio rerio* infection model, OprF is important for *P. aeruginosa* virulence in a macrophage-dependent manner

**Figure 1:**
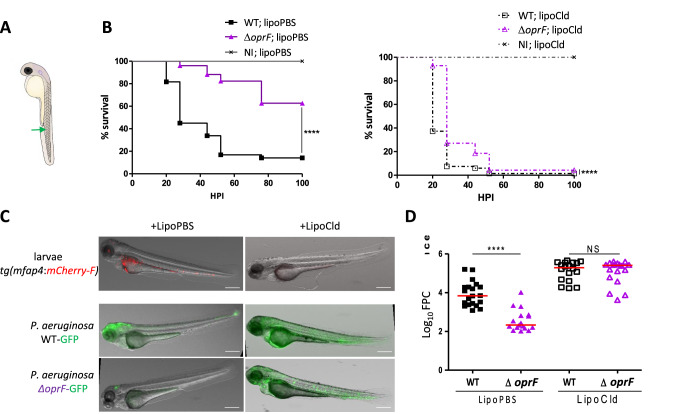
The *P. aeruginosa* Δ*oprF* mutant is attenuated in zebrafish embryos in a macrophage-dependent manner. (**A**) Diagram of 50 h post-fertilization (hpf) zebrafish embryo showing the caudal vein injection site (green arrow). (**B**) Survival curves of embryos infected with PAO1 wild-type stain or PAO1 Δ*oprF* mutant. Non infected embryos (NI) were used as control. Embryos were either treated with LipoPBS (left panel) or with LipoCld (right panel) to deplete macrophages. Approximately 3500 CFU *P*. *aeruginosa* were microinjected into the caudal vein (n = 50–70 for infected embryos and n = 30 for NI embryos, pool of three independent experiments). Results are expressed as the percentage of surviving embryos at different times post-infection. (**C**) Representative fluorescence images of embryos infected by either wild-type or Δ*oprF P. aeruginosa* (at 20 hpi) in the context of LipoPBS or LipoCld treatment. The efficiency of macrophages depletion is shown in the upper panels with the visualization of red macrophages. Scale bar 400 µm. (**D**) Quantification of bacterial loads (fluorescence pixel counts) from two independent experiments. Each symbol represents individual embryo (n = 18–20) and horizontal lines indicate the median values. Statistical significance was determined by log-rank test (**B**) or one-tailed Mann–Whitney’s test (**D**).

We evaluated the role of OprF in *P. aeruginosa* virulence in the zebrafish (*Danio rerio)* embryo model. Zebrafish embryo is a model of choice, which has been used for various intracellular and extracellular bacterial pathogens, to investigate the contribution of innate immune cells during infection^19,20^. In a previous study, we have introduced a plasmid constitutively producing GFP^8^ in an *oprF* mutant in the PAO1 background^11^. Here, the same mutant and the isogenic wild-type strain were injected intravenously in the caudal vein of embryos at 50 h post-fertilization (Fig. 1A). The survival curves of infected embryos indicate that OprF is a critical virulence determinant in this model since the virulence of *oprF* mutant is significantly attenuated as compared to the one of wild-type PAO1 strain (Fig. 1B, left graph). In addition, fluorescence microscopy of infected embryos (20 h post-infection) showed a lower bacterial burden with *oprF* mutant than wild-type strain (Fig. 1C, quantification in Fig. 1D). A similar result was observed when bacterial burden was assessed by colony forming unit (CFU) counting (Fig. S1).

To evaluate the contribution of macrophages in the attenuated phenotype of *oprF* mutant, we took advantage on the fact that macrophages can be depleted from zebrafish embryos with a validated method that uses liposome-encapsulated clodronate (LipoCld)^23,24^. Macrophage-depleted zebrafish embryos have been shown to be highly sensitive to *P. aeruginosa* infection^6,21^. We have carried out experiments with *tg(mfap4:mCherry-F)* embryos where macrophages are visualized as red cells^25^, which allows to check macrophage depletion upon LipoCld injection (Fig. 1C, upper panels). Interestingly, the survival curve of macrophage-depleted embryos showed a dramatic increase in the virulence of *oprF* mutant strain, since all embryos were killed, with a slight delay comparatively to the wild-type strain (Fig. 1B, right graph). Thus, macrophage depletion largely attenuates the difference of survival between wild-type and mutant strain. In agreement with this finding, bacterial burden was elevated and not significantly different for both wild-type and mutant strains in the context of macrophage depletion (Fig. 1C,D).

Taken together, these results indicate that OprF plays a crucial role during acute infection of *P. aeruginosa* in the zebrafish embryo model and that *oprF* mutant strain is better cleared than wild-type strain in a macrophage-dependent manner.

### Visualisation of macrophages in infected zebrafish embryos indicates no major difference in macrophage recruitment or phagocytosis efficiency between wild-type *P. aeruginosa* and *oprF* mutant

**Figure 2:**
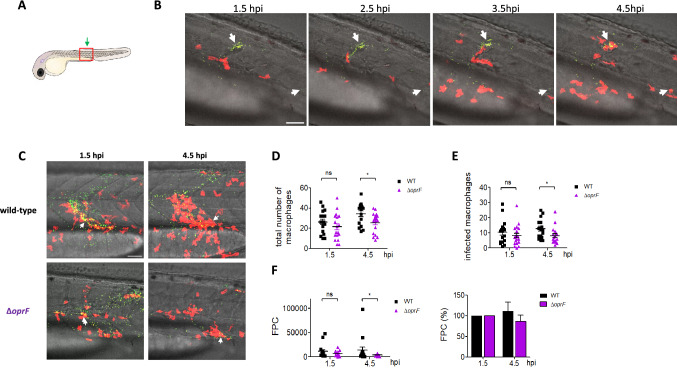
Real-time visualization and quantification of phagocytosis of *P. aeruginosa* after local injection into muscle of wild-type and Δ*oprF* strains. (**A**) Illustration of zebrafish larva with the muscle (in red) injection site (green arrow). (**B**) Confocal time-lapse images of *tg*(*mfap4:mCherry-F*) larva (red macrophages) infected with Δ*oprF P. aeruginosa* (green) by injection in the muscle. The same area was imaged every hour from 1.5 hpi. White arrows depict GFP-expressing *P. aeruginosa* that will be taken up by macrophages, which are recruited at the bacterial location. Maximum intensity projection of 96 sections every 1 µm, scale bar 50 µm. (**C**) Confocal time-lapse images of *tg(mfap4:mCherry-F)* larvae (red macrophages) infected with wild-type or Δ*oprF P. aeruginosa* (green) by injection in the muscle. The left panel is at 1.5 hpi and the right panel a 4.5 hpi. White arrows depict GFP-expressing *P. aeruginosa* phagocytosed by macrophages. Maximum intensity projection of 96 sections every 1 µm, scale bar 50 µm. (**D**) Quantification of recruited macrophages at 1.5 and 4.5 hpi, from three independent experiments. Each symbol represents individual embryo (16 to 18 embryos for each strain) and horizontal lines indicate the mean values ± SEM. Statistical significance was determined by two-tailed *t* test, ns—not significant, *p < 0.05. (**E**) Quantification of infected macrophages, from three independent experiments. Mean ± SEM, two-tailed Mann–Whitney’s test, ns—not significant, *p < 0.05. (**F**) Quantification of bacterial loads (fluorescence pixel counts) from three independent experiments, presented as total value (left) or percentage of the FPC value at first time point (1.5 hpi), counted for each embryo. Mean ± SEM, two-tailed Mann–Whitney’s test, ns—not significant, *p < 0.05.

Confocal microscopy after local injection into closed cavities, such as hindbrain ventricle (HBV) or muscle allows to visualize recruited macrophages and bacteria phagocytosed by macrophages close to the site of injection^26^. Upon HBV infection, wild-type and Δ*oprF* bacteria can be visualized within macrophages 2 h after infection (Fig. S2). A similar finding is observed upon muscle injection. To visualize macrophage recruitment and phagocytosed bacteria in real-time, a time lapse experiment was performed during 3 h. Injection in the muscle was preferred to HBV injection because of easier positioning of embryos and lower thickness that facilitates z-stacks analysis (Fig. 2A). The recruitment of macrophages that phagocytose *P. aeruginosa* can be clearly observed, both for clustered bacteria and isolated bacteria, as shown for Δ*oprF* bacteria (Fig. 2B). The number of recruited macrophages and the clearance of *oprF* mutant by macrophages were then compared to the one of wild-type strain by performing quantification on images from time 1.5 h and 4.5 h on 16 to 18 embryos for each strain (Fig. 2C). The number of recruited macrophages is similar for both strains at time 1.5 h and slightly, but significantly, higher for wild-type strain at 4.5 h (Fig. 2D). Similarly, the number of infected macrophages appeared similar for both strains at time 1.5 h and slightly, but significantly, higher for wild-type strain at 4.5 h (Fig. 2E). In contrast, a slight, but significant, reduction for bacteria counting was observed for the mutant strain comparatively to wild-type strain at 4.5 h (Fig. 2F), indicative of a better clearance of bacteria. This is correlated with a tendency of increased green Fluorescent Pixel Counts (FPC) for wild-type strain and decreased FCP for *oprF* mutant at 4.5 h relatively to FPC at 1.5 h (Fig. 2F, right panel).

Cumulatively, our results revealed that confocal microscopy after local injection into hindbrain ventricle or muscle is a highly suitable approach to visualize and quantify in real-time *P. aeruginosa* phagocytosis by recruited macrophages. The better clearance of the *oprF* mutant does not seem to be linked to an increase in macrophage recruitment or phagocytosis efficiency. To dissect intramacrophage mechanisms underlying *oprF* mutant clearance, we carried out experiments on cultured macrophages.

### *P. aeruginosa oprF* mutant exhibits reduced resistance to cultured macrophages, which is correlated with elevated association of bacteria with acidic compartments

**Figure 3:**
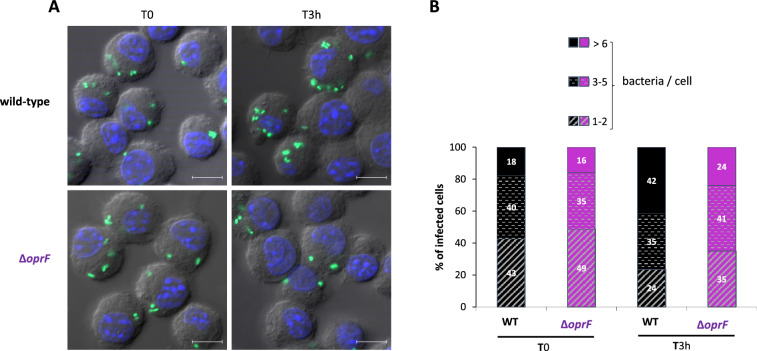
Visualization and quantification of intracellular *P. aeruginosa* within macrophages. GFP-expressing PAO1 WT and ∆*oprF* strains were used for infecting J774 macrophages. Cells were fixed after phagocytosis and 20 min (T_0_) or 2.5 h (T_3h_) treatment with gentamycin and imaged with fluorescent microscope. DAPI was used to stain the nucleus. (**A**) Visualization of intracellular bacteria. The merged image shows Differential Interference Contrast (DIC), nucleus staining (blue) and bacteria expressing GFP (green). The scale bars depict 10 µm. (**B**) Count of the number of bacteria in infected macrophages from images obtained. The numbers of bacteria per cell were classified in three groups (1–2, 3–5 and > 6 bacteria per cell) and percentage of each class is shown. Count is done from 100 cells per condition and results are expressed as means from three independent experiments.

An otopathogenic *P. aeruginosa oprF* mutant strain was found to be more sensitive to macrophage killing than the wild-type strain upon infection of mouse bone marrow macrophages^7^. In a previous study, we have used the *oprF* mutant in the PAO1 background to address the cytotoxicity driven by phagocytosed bacteria in macrophages^8^. Here, we quantified intracellular bacteria after phagocytosis of *P. aeruginosa* strains. J774 macrophages were infected with wild-type PAO1 and *oprF* mutant strains expressing constitutively GFP grown exponentially in LB medium (Multiplicity of infection or MOI = 10). After 25 min of phagocytosis, several washes were performed to remove adherent bacteria and gentamicin was added to kill extracellular bacteria. Microscopic observation of infected macrophages was done after 20 min or 2.5 h of gentamicin treatment (Fig. 3A). The number of bacteria per macrophage was quantified and classified in three groups (Fig. 3B). Wild-type and mutant strains behaved similarly at the early time point. At the latest time, macrophages infected with *oprF* mutant were found to harbor less bacteria than macrophages infected with wild-type strain. This result supports a role for OprF to limit the elimination of *P. aeruginosa* PAO1 by macrophages.

**Figure 4:**
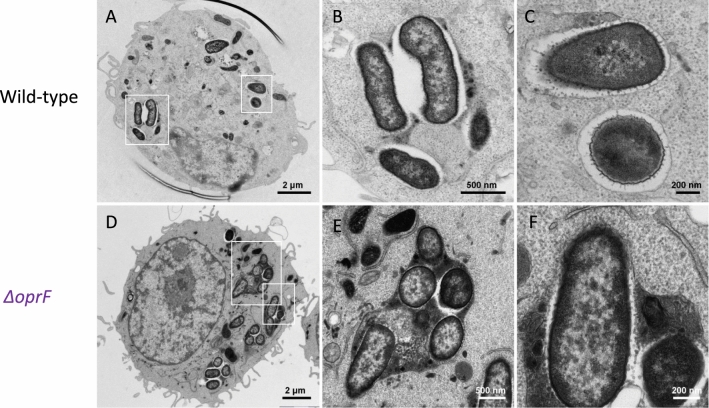
Transmission electron micrographs (TEM) of *P. aeruginosa* within macrophages. J774 macrophages were infected with *P. aeruginosa* wild-type strain or *oprF* mutant for 50 min after phagocytosis and subjected to TEM to visualize intracellular bacteria. For the wild-type strain, most of bacteria were found inside membrane bound vacuoles (**A**–**C**) whereas *oprF* mutant was mostly found in phagolysosomes (**D**–**F**). (**B**) and (**C**) are showing details of the cell shown in (**A**) (white rectangles) at higher magnification, (**E**) and (**F**) show details of the cell shown in (**D**) (white rectangles).

We further examined intracellular *P. aeruginosa oprF* mutant within macrophages in more detail using transmission electron microscopy (TEM). J774 macrophages infected with wild-type PAO1 strain or *oprF* mutant were subjected to fixation at different time points after phagocytosis. At early time point (50 min post phagocytosis) intracellular wild-type *P. aeruginosa* are essentially observed in membrane bound vacuoles (Fig. 4A–C). For the *oprF* mutant, bacteria were frequently observed in vacuoles partially or totally filled with heterogeneous electron dense material, suggesting that the vacuoles had fused with lysosomes (Fig. 4D–F). At time 2.5 h post phagocytosis, intracellular bacteria were rarely identifiable for the *oprF* mutant, suggesting that most of them have been destroyed, while for the wild-type strain, numerous bacteria were observed in clear vacuoles or in the cytoplasm with no surrounding membrane as previously described^8^. In the course of TEM analysis of J774 macrophages infected by wild-type PAO1 strain, we noticed the presence of vesicles in the vacuolar space along the phagosomal membrane (Fig. 4C,F). These vesicles seemed issued from bacteria and were often lying along the phagosomal membrane (Fig. S3, A-B). Because OprF is a major component of outer membrane vesicles (OMVs)^27^, we investigated whether such bacterial vesicles were also present in the *oprF* mutant. Vesicles were clearly produced from *oprF* mutant as well and found associated with the phagosomal membrane (Fig. S3, C-D).

**Figure 5:**
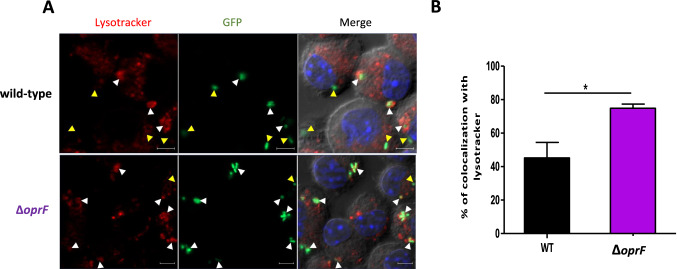
Colocalization of *P. aeruginosa* with a probe that labels acidic compartments. J774 macrophages were infected with PAO1 and ∆*oprF* strains expressing GFP. After 2.5 h of gentamicin treatment, infected J774 cells were incubated with Lysotracker for 10 min, a red fluorescent weak base that accumulates in acidic compartments. Cells were then fixed and imaged with fluorescence microscope. (**A**) The image shows individual panels for Differential Interference Contrast (DIC), lysosomal compartment (red), bacteria expressing GFP (green), the nucleus (blue) and merged image of all channels. White arrows show colocalization of bacteria with lysotracker while yellow arrows show non-colocalization. Scale bar is equivalent to 5 µm. (**B**) Values indicate the percentage of bacteria that colocalized with Lysotracker red (white arrows). Data are the mean (± SEM) of 3 independent experiments, with a minimum of 100 bacteria counted per experiment for each sample. *, *p* ≤ 0.05 (Two-tailed unpaired *t* test).

To quantify the association between bacteria and acidic compartments such as phagolysosomes, we examined the association between fluorescent PAO1 wild-type and *oprF* mutant bacteria and the LysoTracker probe during infection using fixed macrophages. Bacteria colocalizing with LysoTracker could be visualized (Fig. 5A) and quantified (Fig. 5B). A significantly higher percentage of bacteria colocalizing with LysoTracker red marker was observed in macrophages infected with *oprF* mutant (~ 75%) comparatively to wild-type strain (~ 45%). This increased localization of *oprF* mutant in acidified compartments corroborates the TEM observation of a preferential localization of Δ*oprF* bacteria in phagolysosomes.

Taken together, this results indicate an intramacrophage survival defect of *oprF* mutant, which is associated with a preferential localization within acidified compartments.

### Contribution of phagosomal acidification to the outcome of infection in vivo

**Figure 6:**
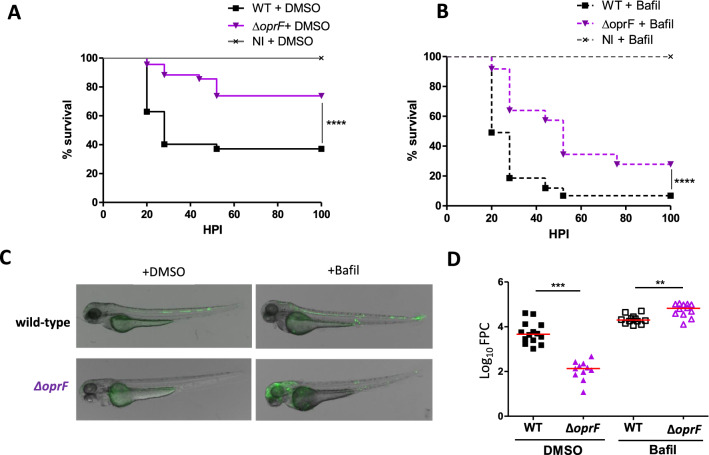
Effect of bafilomycin on the virulence of wild-type and Δ*oprF* mutant in zebrafish embryos. Embryos were infected with either wild-type or Δ*oprF P. aeruginosa* (2000–2500 CFU) in the hindbrain ventricle at 2 dpf or not infected (NI) (n = 60–70 for infected embryos and n = 45 for NI embryos, pool of three independent experiments). Survival curves of embryos treated with 50 nM bafilomycin (Bafil) (**B**) or treated with DMSO as control (**A**). Statistical significance was determined by log-rank test. Imaging (**C**) and quantification (**D**) of bacterial loads (fluorescence pixel counts) representative of two independent experiments at 20 hpi (n = 11–14). Each symbol represents individual embryo and horizontal lines indicate the median values. Statistical significance was determined by one-tailed Mann–Whitney’s test.

Because *oprF* mutant attenuation in zebrafish is dependent on the presence of macrophages and because the *oprF* mutant colocalizes more frequently than wild-type strain with acidic compartments in infected macrophages, we hypothesized that decreasing phagosomal acidification in zebrafish embryos may alleviate the attenuation of *oprF* mutant. The acidification of the phagosome is dependent on the activity of the host vacuolar ATPase, which can be specifically inhibited by the inhibitor bafilomycin A1^28^. Bafilomycin was added to the larvae bath water as reported earlier^29^ to test the implication of macrophage phagosomal acidification as a microbicidal mechanism responsible for the attenuated phenotype of *oprF* mutant in zebrafish larvae. The survival of embryos infected with wild-type strain and *oprF* mutant was reduced in the presence of bafilomycin, supporting the idea that phagosomal acidification is important for host defense (Fig. 6A,B). We reasoned that if increased elimination by acidified phagosomes contributes to *oprF mutant* attenuation, then bafilomycin treatment should suppress this attenuated phenotype. This is partially the case, since the virulence of *oprF* mutant is greatly increased in the presence of bafilomycin (Fig. 6B). The bacterial burden was evaluated by microscopy analysis and quantification of the FPC (Fig. 6C). Bacterial fluorescence counts are significantly lower for *oprF* mutant than wild-type strain upon HBV injection, which is correlated with lower CFU (Fig. S4), and is consistent with results upon caudal vein injection (Fig. 1D). In contrast, the number of fluorescent pixels for the *oprF* mutant was strongly increased in the presence of bafilomycin, becoming significantly higher than the one of wild-type strain (Fig. 6C,D). These data support the hypothesis that acidification contributes to limit the growth of *oprF* mutant in non-treated embryos.

Cumulatively, results carried out with cellular and animal models indicate that OprF acts by protecting *P. aeruginosa* against macrophages clearance during acute infection, in part by avoiding acidified phagosomes.

## Discussion

Recent studies support the ability of *P. aeruginosa* to reside, at least transiently, in macrophages and bacterial factors that play a role in the intramacrophage stage, such as OprF, are starting to be identified. However, their role towards macrophage bactericidal action remains mostly elusive. In the present study, we have taken advantage of the zebrafish embryo model, in combination with a cellular infection approach, and *orpF* mutant to better understand how *P. aeruginosa* resists to macrophage clearance during acute infection.

We have evaluated for the first time the role of OprF in *P. aeruginosa* virulence in a vertebrate model harbouring functional macrophages, the zebrafish embryo model. This model, which has been used for various intracellular and extracellular bacterial pathogens, is a model of choice to investigate the contribution of innate immune cells during infection^20^. Zebrafish embryo is increasingly considered for modeling human infections caused by bacterial pathogens, including those affecting the lungs^30,31^. Moreover, *P. aeruginosa* bacteria have been visualised within macrophages upon zebrafish embryo infection^6,21,22,32,33^, suggesting a relevance for this model to evaluate the role of intramacrophage step in the outcome of infection. Our results showed an attenuation of the *oprF* mutant in zebrafish embryos, which is dependent on the presence of macrophages, thus supporting the importance of OprF in resistance to macrophage. Injection of bacteria into closed cavities, such as hindbrain ventricle, or into muscle provides an outstanding opportunity to visualize macrophage recruitment and bacterial phagocytosis in real-time^26^. Images of infected embryos clearly support the importance of macrophages in *P. aeruginosa* clearance, with a large proportion of bacteria being engulfed by macrophages. Based on measurement of bacterial load, the *oprF* mutant appeared to be cleared more efficiently than wild-type strain. However, it was not associated with higher recruitment of macrophages or higher phagocytosis rate of the *oprF* mutant strain by macrophages in vivo. The ability of OprF to bind the complement component C3b, which tags bacteria for phagocytosis by host macrophages and neutrophils, was previously proposed to be responsible for a reduced internalization by neutrophils after opsonization of *oprF*-deficient bacteria compared with wild-type *P. aeruginosa*^34^. Because the internalization within macrophages does not seem to be impacted in our study, the behavior of *oprF* mutant strain in zebrafish embryos is unlikely linked to the complement system, possibly due to the fact that bacteria are not opsonized.

OprF has been previously involved in the intramacrophage survival otopathogenic of *P. aeruginosa*^7^. Upon phagocytosis in cultured macrophages, *P. aeruginosa* first resides in membrane bound vacuoles, whereas a cytosolic location can be observed at later time of infection^7,8^. Moreover, ruptured phagosomal membrane has been visualized by TEM in macrophages infected with wild-type *P. aeruginosa* and a quantitative phagosomal rupture assay has revealed that *oprF* mutant is less efficient to escape from phagosome than wild-type PAO1 strain^8^. Here, we showed that *oprF* mutant in the PAO1 genetic background exhibited a higher association with acidic compartments of cultured macrophages, identified as phagolysosomes by TEM, than the wild-type strain. In addition, a lower number of intracellular bacteria per macrophage was visualized 3 h after infection with the mutant strain compared to wild-type, thus supporting a correlative link between preferential colocalization with acidified compartments and intramacrophage survival defect for *oprF* mutant. Importantly, the *oprF* mutant recovered virulence in zebrafish embryo upon treatment with bafilomycin, known to hinder acidification, thus supporting the hypothesis that OprF acts by facilitating bacterial avoidance to macrophage acidification.

The outer membrane protein OprF is involved is cell envelope integrity and abundant amounts of OprF are present in *P. aeruginosa* outer membrane vesicles (OMVs), from both planktonic cell supernatants and biofilms^13,27^. In the present study, TEM allowed us to visualize vesicles that are likely of bacterial origin found associated with the phagosomal membrane. The intracellular function, if any, of these vesicles is unknown. Vesicles were also clearly produced from *oprF* mutant as well, which is consistent with the fact that while OprF is abundant in OMVs, an *oprF* mutant was shown to produce more OMVs than wild-type in liquid culture^35^. Thus, we concluded that such vesicles should not be linked to the phenotype of *oprF* mutant intracellularly, even though we cannot exclude functional differences between OMVs from wild-type strain and *oprF* mutant.

OprF plays also an important role in the regulation of *P. aeruginosa* virulence factors. Notably, *P. aeruginosa oprF* mutant has been shown to reduce expression of T3SS genes and secretion of ExoT and ExoS toxins^6,11^, which are bi-functional cytotoxins that contain N-terminal RhoGAP domains and C-terminal ADP-ribosylation domains^36^. Interestingly, similarly to the *oprF* mutant, a T3SS mutant was attenuated in zebrafish embryos and macrophage depletion restored the virulence of the attenuated T3SS strain^21^. The in vivo phenotype of *oprF* mutant could therefore be related to a reduced expression of T3SS genes. Moreover, the phagosomal escape defect of *oprF* mutant has been attributed to the negative regulation of T3SS expression inside macrophages, since T3SS and more specifically ExoS were found to play a role *P. aeruginosa* phagosomal escape^8^. Hence, the stronger association of *oprF* mutant with acidified compartments may reflect bacterial location in phagolysosome due to a lower ability to escape from the phagosome into the cytosol, in relation with the decreased expression of T3SS genes and reduced secretion of ExoS toxin. Alternatively, OprF, and the T3SS effector ExoS, could play a role in limiting phagolysosomal fusion or acidification of the phagosome. Remarkably and consistent with our findings with macrophages, *P. aeruginosa* has been proposed to utilize ExoS to avoid acidified compartments within epithelial cells^37^. Whether ExoS inhibits directly vacuolar acidification in epithelial cells directly, or by redirecting bacteria to other compartments within the cell was not determined.

In summary, based on a combination of animal and cellular experimental approaches, our results indicate that OprF protects *P. aeruginosa* against macrophage clearance during acute infection, by avoiding destruction in phagolysosomes. The mechanism involved in the avoidance of acidic compartments is not characterized and could involve a facilitation of phagosome escape and/or an impairment of phagosome maturation. Based on previous work, this effect is likely correlated with the intracellular effect of OprF on expression of ExoS, a T3SS effector which has been implicated in phagosomal escape^8^ and avoidance of acidified compartments in epithelial cells^37^. Our results also highlight the contribution of macrophages in the clearance of *P. aeruginosa* during acute infection in the zebrafish embryo model, which appears as the state-of-the-art model to address in vivo the role of macrophages during *P. aeruginosa* infection.

## Materials and methods

### Bacterial strains and growth conditions

Bacterial strains and plasmids are described in Table 1. *P. aeruginosa* was grown at 37 °C in Luria broth (LB). Plasmid pMF230 expressing GFP constitutively^38^ (obtained from Addgene) was introduced in *P. aeruginosa* by conjugation, using an *E. coli* strain containing pRK2013. Recombinant bacteria were selected on LB agar plates containing carbenicillin (300 μg/ml) and triclosan (15 µg/ml).

**Table 1:** Bacterial strains and plasmids used in the study.

Name	Description	References
H103 PAO1	Wild-type	^11^
H636	Δ*oprF*	^11^
pRK2013	Tra^+^, Mob^+^, ColE1, Km^r^	Laboratory collection
pMF230	GFP*mut2,* Amp^r^	^38^

### Infection of *Danio rerio* embryos

Experiments were performed using the AB zebrafish or the *tg(mfap4:mCherry-F)* zebrafish line harboring red-fluorescent macrophages^25^ and maintained under standard conditions^6^. Bacterial strains, which were freshly streaked out from glycerol stocks, were grown in LB medium to mid-log phase (DO = 0.7 to 0.8), recovered by centrifugation and washed twice in Phosphate-Buffered Saline (PBS). Suspensions were homogenized through a 26-gauge needle and resuspended in PBS at about 10^9^ bacteria/ml added with 10% phenol red to aid visualization of the injection process. Infection were carried by the direct microinjection of 2 nl of bacterial suspensions into the caudal vein of 50 hpf embryos, previously dechorionated and anesthetized with 0.02% tricaine. For survival kinetics after infection, the number of dead embryos was determined visually based on the absence of heartbeat. For phagocytosis visualization, 1500–2000 CFU were injected locally into the hindbrain vesicle (HBV) or the muscle of 50 hpf *tg(mfap4:mCherry-F)* larvae. To quantify bacteria by CFU counting, infected larvae were dissolved by pipetting in 200 μL 1% PBS 1X-Triton solution at 20 hpi, and plated on LB plates containing ampicillin (100 μg/ml). Depletion of macrophages was carried out upon microinjection of LipoCld or lipoPBS as control into the caudal vein of 24–30 hpf embryos zebrafish^24^ and visualized by fluorescence microscopy. To inhibit acidification by host vacuolar ATPase, larvae were treated with Bafilomycin A1 (Interchim) at 50 nM in 0.5% DMSO via soaking 30 min before infection in the HBV, as described above^29^.

### Microscopic analysis of zebrafish embryos, quantification of bacterial load by fluorescent pixel counts and quantification of recruited/infected macrophages

For live imaging, anesthetized infected embryos were mounted in 35 mm glass-bottom dishes (FluoroDish, World Precision Instruments, UK) and immobilized with 1% low-melting point agarose. Direct visualization is performed as before^6^ using an Olympus MVX10 epifluorescent stereomicroscope equipped with a digital color camera (Olympus XC50). Fluorescence and bright-field images are acquired and processed with CellSens (Olympus) and assembled using GIMP 2.6 freeware and Image J software to adjust contrast and brightness and to remove out-of-focus background fluorescence.

For phagocytosis observation, immobilized embryos were immersed with fish water containing tricaine for direct visualization using spinning disc Nikon Ti Andor CSU-W1 microscope (40x/1.15 Water objective).

For time lapse video microscopy of *Pseudomonas* /macrophage interaction, we used as before^39^ an ANDOR CSU-W1 confocal spinning disk on an inverted NIKON microscope (Ti Eclipse) with ANDOR Neo sCMOS camera (40 × NA 1.15 water objective). Image stacks for time-lapse movies were acquired at 28 °C every hour, typically spanning 120 μm at 1–2 μm intervals. The 4D files generated from time-lapse acquisitions were processed using Image J, compressed into maximum intensity projections. For quantification of bacterial load by Fluorescent Pixel Counts (FPC), fluorescent bacteria were injected in the larvae and imaged using MVX10 Olympus stereomicroscope or using maximum projection from confocal stacks. Fluorescence was quantified as before^39^ by computation using Fiji (ImageJ software) as following: 1/ Background was measured in images of PBS injected larvae and then was subtracted in the fluorescence images, 2/ “make binary” function was run, and 3/ “measure area” function was used to determine the number of fluorescent pixels of the image, with avoiding the auto-fluorescence of the yolk.

Recruited and infected macrophages were counted manually from z-stacks of obtained microscopy images, using Fiji (ImageJ) software, after the brightness/contrast adjustment for better visualization. Macrophages were considered as infected if macrophage fluorescence overlaps with bacterial fluorescence in respective z-stack.

### Infection of cultured macrophages and visualization and quantification of intracellular bacteria by fluorescent microscopy

J774A.1 cells were maintained at 37 °C in 5% CO_2_ in Dulbecco's modified Eagle medium (DMEM) (Gibco) supplemented with 10% fetal bovine serum (FBS) (Gibco). The infection of J774 macrophages seeded on glass coverslips by *P. aeruginosa* was carried out as described previously^6^. After 2.5 h of gentamicin treatment, the cells were washed twice with PBS and fixed with 4% paraformaldehyde for 30 min. To visualize acidified compartments, macrophages were incubated with 50 nM Lysotracker red DND-99 (Molecular Probes) in DMEM (supplemented with 10% FBS) for 10–15 min before fixation to stain lysosomes exclusively. After fixation, cells were washed, mounted on glass slides in Vectashield with DAPI (Vector Laboratories, Inc) and slides were examined as described previously^8^ using an upright fluorescence microscope (Axioimager Z2, Zeiss) equipped with an Apotome 1 for optical sectioning. A 63X Apochromat Objective (NA 1.4). Transmitted light was acquired using differential interference contrast (DIC), Fluorescein isothiocyanate (FITC) filter was used to visualize GFP expressing bacteria and Lysotracker red fluorescence was acquired using a texas red filter set. Images were processed using ZEN blue software (Zeiss).

### Transmission electron microscopy

Macrophages were seeded on glass coverslips and infected as described above. Infected cells were fixed with 2.5% gluteraldehyde and treated as described previously^8^. Dehydration was performed through acetonitrile series and samples impregnated in epon 118: acetonitrile 50:50, followed by two times for 1 h in 100% epon, were treated as described^8^. Ultrathin sections of 70 nm were cut with a Leica UC7 ultramicrotome (Leica microsystems), counterstained with uranyl acetate and lead citrate and observed in a Jeol 1200 EXII transmission electron microscope. All chemicals were from Electron Microscopy Sciences (USA) and solvents were from Sigma. Images were processed using Fiji software.

### Ethics statement

All animal experiments described in the present study were conducted at the University of Montpellier according to European Union guidelines for handling of laboratory animals (http://ec.europa.eu/environment/chemicals/lab_animals/home_en.htm) and were approved by the Direction Sanitaire et Vétérinaire de l'Hérault and Comité d'Ethique pour l'Expérimentation Animale under reference CEEA-LR-B4-172-37 and APAFIS#5737-2016061511212601 v3. The breeding of adult fish adhered to the international guidelines specified by the EU Animal Protection Directive 2010/63/EU and adult zebrafish were not sacrificed for this study. All experiments were performed before the embryos free-feeding stage and did not fall under animal experimentation law according to the EU Animal Protection Directive 2010/63/EU. For survival curves, cardiac rhythm was used as a clinical criterium. Embryos were euthanized using the anaesthetic Tricaine up to a lethal dose (500 mg/ml) before bleach treatment.

### Statistical analysis

Statistical analyses for ex vivo experiments with J774 cells was performed using *t*-test and comparisons between survival curves were performed using the log rank test with Prism 5.01 (GraphPad, Inc.). Statistical analysis for macrophage recruitment and phagocytosis is indicated in the legend of Fig. 2. In the figures, * means *p* value ≤ 0.05, ** ≤ 0.01, *** ≤ 0.001 and **** ≤ 0.0001.

## Supplementary Information

Supplementary Information
