# Supplementary material for: Pseudomonas aeruginosa OprF plays a role in resistance to macrophage clearance during acute infection
Source: Sci Rep. 2021 Jan 11;11:359. doi: 10.1038/s41598-020-79678-0 (PMC7801371; doi:10.1038/s41598-020-79678-0)
Supplement: Supplementary file 1 — Supplementary Information [file 41598_2020_79678_MOESM1_ESM.pdf]

Pseudomonas aeruginosa OprF plays a role in resistance to macrophage clearance during acute infection

Malika Moussouni[1], Laurence Berry[1$], Tamara Sipka[1$], Mai Nguyen-Chi[1] and Anne Blanc-Potard[1*]

Supplementary Figure 1

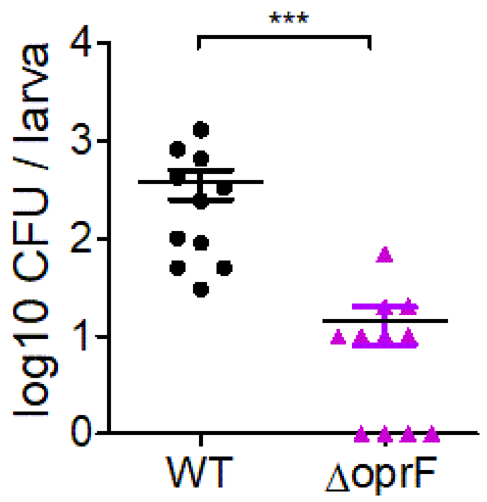

Quantification of bacteria by CFU after intravenous injection. Quantification of Colony Forming Unit (CFU) at 20 hpi within larvae infected intravenously with the 3500 CFU of wild-type bacterial strain (WT) and *oprF* mutant (n = 12 for each strain). Statistical significance was determined by two-tailed Mann-Whitney's test, ***$p<0.001$. Outliers were determined using Grubbs' test (Graph Pad Prism 4.0 Software) and removed from the analysis (1 in each group).

Supplementary Figure 2

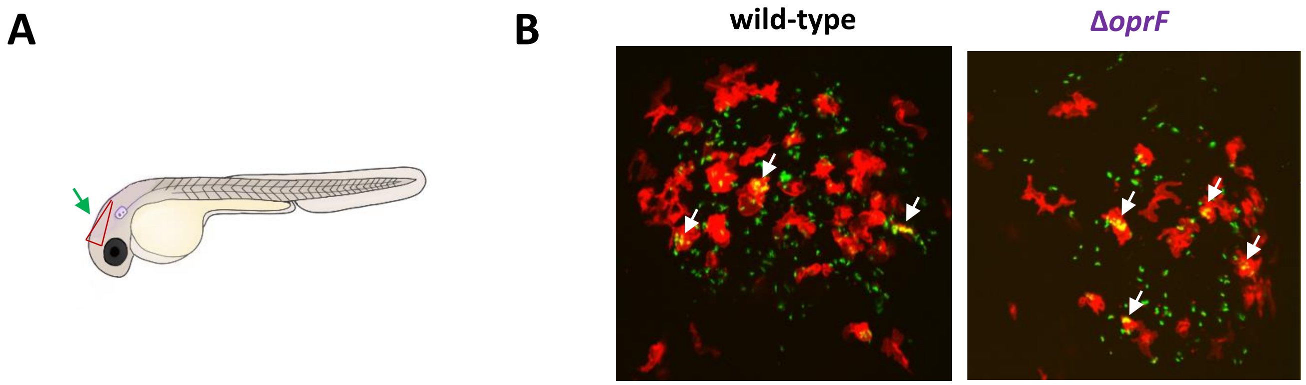

Visualization of phagocytosis of *P. aeruginosa* after local injection into hindbrain ventricle. (A) Illustration of zebrafish larva with the hindbrain ventricle (in red) injection site outlined in green. **(B)** The hindbrain ventricle is accessible to recruited myeloid cells and the phagocytosis of *P. aeruginosa* strains can be visualized using tg*(mfap4:mCherry)* embryos that harbor red macrophages. Hindbrain ventricle injection of wild-type or Δ*oprF* GFP-expressing *P. aeruginosa* was imaged at 2 hpi and white arrows depict phagocytosed bacteria. Confocal microscopy was performed with maximum intensity projection of 64 sections every 1 µm, scale bar 30 µm.

Supplementary Figure 3

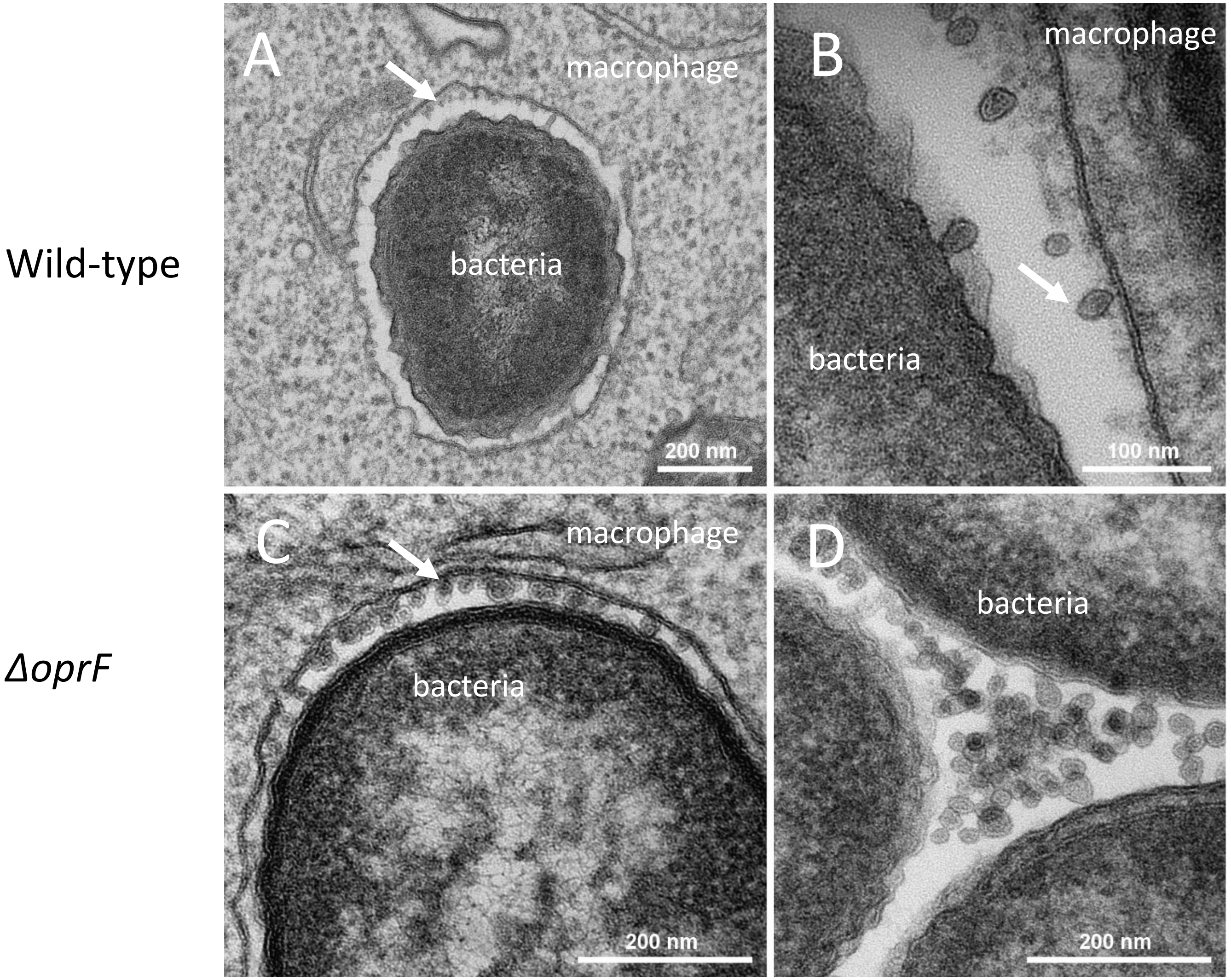

Visualization of bacterial vesicles in infected phagosomes. TEM analysis denoted the presence of vesicles issued from bacteria in PAO1 WT **(A-B)** and Δ*oprF* strain **(C-D)**. These vesicles appeared often stuck to the vacuole membrane (white arrows).

Supplementary Figure 4

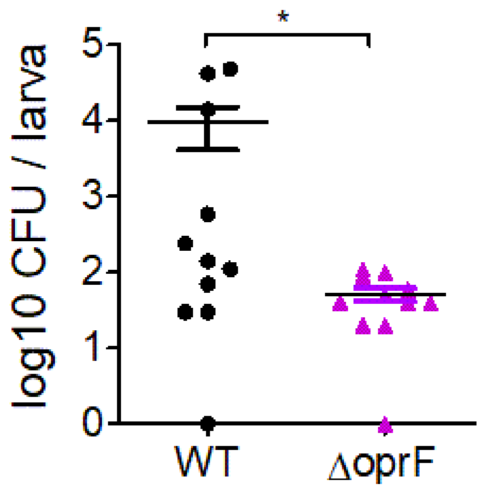

Quantification of bacteria by CFU after HBV injection. Quantification of Colony Forming Unit (CFU) at 20 hpi within larvae injected in HBV with 2100 CFUs of wild-type bacterial strain (WT) and *oprF* mutant (n = 12 for each strain). Statistical significance was determined by two-tailed Mann-Whitney's test, *p<0.05. Outliers were determined using Grubbs' test (Graph Pad Prism 4.0 Software) and removed from the analysis (1 in each group).
